# *Escherichia coli* Sequence Type 131-H22 in Parrots from Illegal Pet Trade, Brazil, 2024

**DOI:** 10.3201/eid3110.241279

**Published:** 2025-10

**Authors:** Victoria Galdino Pavlenco Rocha, Fernanda Borges Barbosa, Joaquim Ruiz, Henrik Christensen, Terezinha Knöbl

**Affiliations:** University of São Paulo, São Paulo, Brazil (V.G.P. Rocha, F.B. Barbosa, T. Knöbl); Universidad Científica del Sur, Lima, Perú (J. Ruiz); University of Copenhagen, Nørregade, Denmark (H. Christensen).

**Keywords:** ExPEC, *Escherichia coli*, ST131-H22, high-risk, bacteria, bacterial infections, APEC, zoonotic lineage, zoonoses, parrots, Brazil

## Abstract

*Escherichia coli* sequence type 131:H:22 is a consequential lineage of extraintestinal pathogenic *E. coli*, associated with human pyelonephritis and sepsis. We report the transmission of avian pathogenic *E. coli* in a parrot rehabilitation center in Brazil and the presence of a high-risk zoonotic lineage of extraintestinal pathogenic *E. coli* sequence type 131-H22.

The regulation of the international wildlife trade is critical for the conservation of species and, according to the 2022 World Wildlife Trade Report (https://cites.org/sites/default/files/common/docs/Pilot_World_Wildlife_Trade_Report_for_CITES_CoP19.pdf), generates an annual revenue of USD ≈220 billion. However, in most countries, there is still an illegal wildlife trade that affects biodiversity, risks the extinction of some species, and leads to transmission of zoonotic diseases. According to the Convention on International Trade in Endangered Species of Wild Fauna and Flora, during 2011–2020, birds occupied the second place of most trafficked genera, behind only plants (*1*). Parrots are one of the most frequently sold birds in Brazil, and confiscated birds can act as reservoirs of bacteria such as avian pathogenic *Escherichia coli* (APEC) (*2*,*3*). In this article, we investigated the transmission of APEC in a group of 19 psittacine birds in Brazil.

## The Study

Fifteen blue-fronted Amazon parrots (*Amazona aestiva*) and 4 macaws (*Ara ararauna*) were confiscated in the state of São Paulo, Brazil, by law enforcement authorities and kept for 4 weeks in a rescue center. At the rescue center, fecal samples were collected at intervals of 5 days. After enriching the samples in brain–heart infusion broth (BD, https://www.bd.com), we cultured 20 μL of the broth on MacConkey agar (BD) at 37°C for 24 hours. We then conducted biochemical identification on the isolates. We screened *E. coli* strains by using PCR for minimal predictor virulence genes (*ompT*, *iss*, *hlyF*, *iutA*, and *iroN*), as described previously (*4*). This study was approved by the Ethics and Animal Use Committee of the School of Veterinary Medicine and Animal Science from the University of São Paulo (approval no. 1572080922).

We detected a total of 9 APEC strains. The transmission dynamic revealed that 1 parrot was colonized by APEC on day 1 (the day of seizure). One macaw became infected 5 days after entering the rescue center. After 30–35 days, 6 parrots and 1 macaw were positive for APEC. Next, we screened the strains for high-risk APEC, as proposed previously (*5*). We conducted whole-genome sequencing on 1 positive strain (RG299.1, isolated from an *A. aestiva* (parrot) by using MiSeq (Illumina, https://www.illumina.com). We used the Galaxy Europe platform (https://usegalaxy.eu) to analyze the genome and assess the quality of raw data and assemblies. To determine the sequence typing, plasmids, and virulence genes, we used the ResFinder (https://genepi.food.dtu.dk/resfinder), PlasmidFinder (https://cge.food.dtu.dk/services/PlasmidFinder), and Virulence Finder (https://cge.food.dtu.dk/services/VirulenceFinder) databases.

For the phylogenetic analysis, we selected *E. coli* sequence type (ST) 131 O25:H4 genomes available on EnteroBase (https://enterobase.warwick.ac.uk/species/ecoli) and compared with the genome of the RG299.1 strain from our study. We included 63 genomes in total. We aligned single nucleotide polymorphisms by using snippy core v4.6.0. (https://github.com/tseemann/snippy). We inferred the tree by using IQ-Tree v2.1.2 (https://iqtree.github.io) to perform neighbor joining with 1,000 bootstraps and rooted them at the midpoint. We conducted visualization and metadata analysis by using iTOL (https://itol.embl.de). The RG299.1 strain showed a close relation with uropathogenic *E. coli* (UPEC) (Figure).

**Figure:**
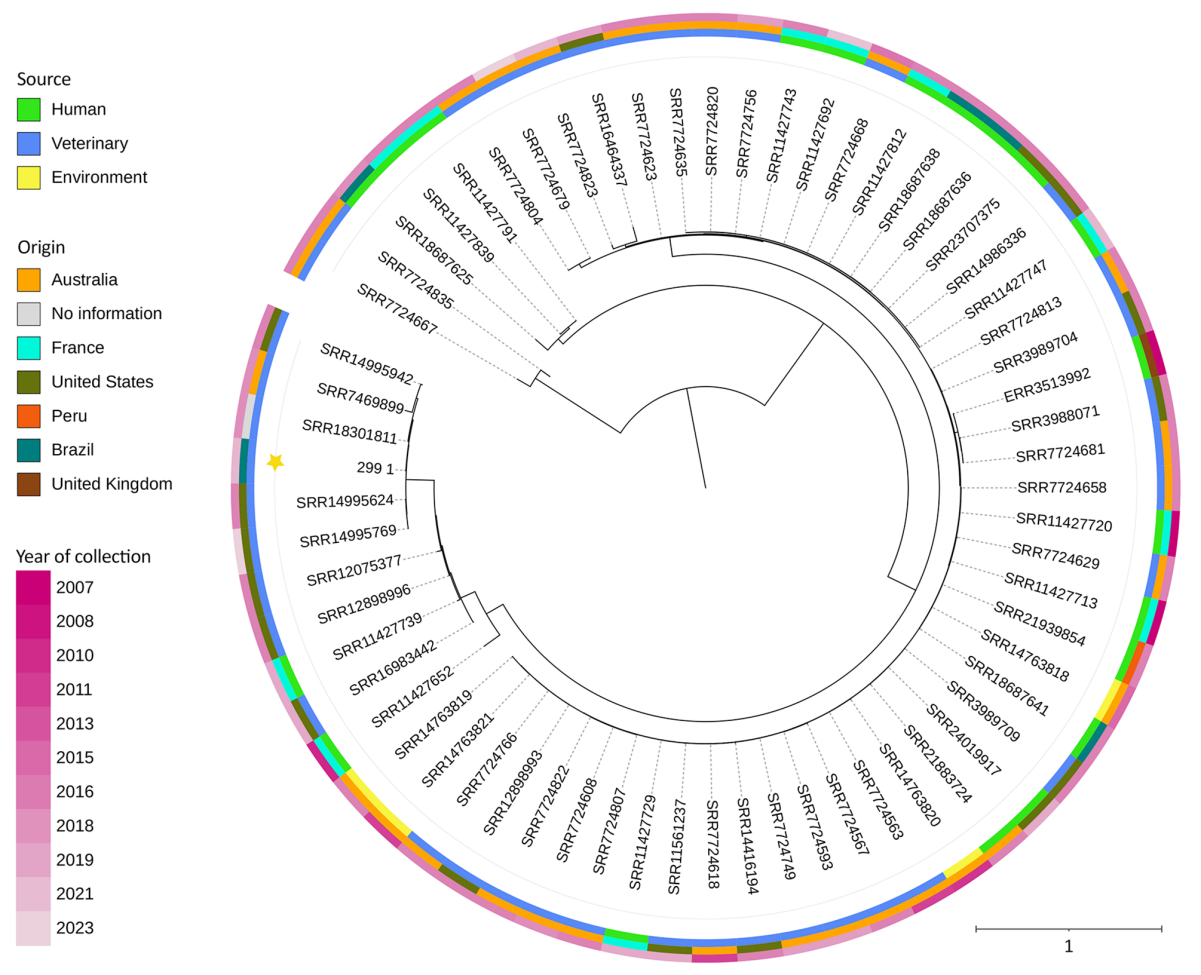
Phylogenetic analysis through a phylogenetic tree of *Escherichia coli* sequence type (ST) 131 isolated from different origins. The tree is rooted at the midpoint and generated to compare the studied strain with public genomes available in EnteroBase (https://enterobase.warwick.ac.uk), connecting 63 strains of ST131 *E*. *coli*. The subtypes from the database are subdivided into the avian and uropathogenic extraintestinal pathogenic *E. coli* pathotypes, along with their origin and the continent where they were isolated and described. The tree shows closely related strains from veterinary origins in South America and Oceania. Strain 299 from this study forms a monophyletic group with 5 sequences from GenBank (accession nos. SRR14995924, SRR7469899, SRR18301811, SRR14995624, and SRRR144995769). This group is related to 3 uropathogenic *E. coli*. strains of human origin in Europe (GenBank accession nos. SRR11427839, SRR11427652 and SRR11427791).

Sequencing confirmed that the study RG299.1 strain (GenBank accession no. SRR31896384) belongs to ST131 phylogroup B2 and serogroup O25:H4. Further characterization of the RG299.1 strain identified allele 22 of the *fimH* type 1 fimbrial adhesin gene (*H22*), confirming that strain belongs to ST131-H22 sublineage (*6*). Currently, ST131-H22 is described as a pandemic lineage of *E. coli*, drawing attention because of high levels of adaptation and resistance to multiple drugs, including fluoroquinolones, betalactams, and carbapenems (*7*,*8*). The RG299.1 strain did not have an antimicrobial resistance profile, possibly because of lower exposure to antimicrobial drugs in the environmental, compared with nosocomial isolates. PlasmidFinder revealed the presence of incompatibility groups IncFIB and IncFII_1 plasmid, but nontransferable mechanisms of resistance were also found. Only chromosomal genes correlated with antimicrobial resistance were present, such as the efflux pump encoder *mdfA*, which can result in quinolone resistance when associated with other acquired genes (*9*).

The RG299.1 ST131-H22 strain carried virulence genes associated with iron transport (*fpeA*, *entS*, *iutA*, *iroN*, and *fepG*), invasion (*ibeA* and *ompA*), and others related to adherence and pathogenicity, such as type 1 fimbriae genes (*fimA*, *fimB*, *fimF*, *fimG*, and *fimH*) (Table). The parrot colonized by strain RG299.1 on the day of seizure died after 15 days. Although the cause of the bird's death was not investigated, it is of note that the presence of virulent strains of *E. coli* in the intestines of Psittaciforme birds is considered undesirable because the microbiota of those birds typically consists of gram-positive bacteria (*3*).

**Table:** Virulence and resistance data of avian pathogenic *Escherichia coli* RG2991.1 ST131 strain from parrots in Brazil, encoding genes and their products found in PlasmidFinder, VirulenceFinder, and ResFinder databases*

Product	Gene or plasmid	Virulence association
Mobility elements	*IncFIB*, *IncFII_1*	Plasmid
Enterobactin components	*entD*, *fepA*, *fes*, *fepC*, *fepG*, *fepD*, *entS*, *fepB*, *entE*, *entF*, *iutA*, *iucD*, *iucC*, *iucB*, *iucA*	Iron acquisition
Iron transport	*chuS*, *chuA*, *chuT*, *chuX*, *chuU*	
Yersiniabactin siderophore biosynthetic protein	*ybtE*, *ybtT*, *ybtU*, *irp1*, *irp2*, *fyuA*	
Salmochelin siderophore system	*iroB*, *iroC*, *iroD*, *iroE*, *iroN*	
K1 capsule	*kpsD*, *kpsT*, *kpsM*	Capsule
General secretion proteins	*gspM*, *gspL*, *gspK*, *gspJ*, *gspI*, *gspH*, *gspG*, *gspF*, *gspE*, *gspD*, *gspC*	General secretory pathway, type II secretion system
Outer membrane protein A	*ompA*	Invasion
Fimbrial proteins	*fimA*, *fimI*, *fimC*, *fimD*, *fimF*, *fimG*, *fimH*, *fimE*, *fimB*	Adhesins, biofilms
Fimbrial adhesins	*yagW/ecpD*, *fdeC*	
Invasion protein	*ibeA*	Invasion

*PlasmidFinder, https://cge.food.dtu.dk/services/PlasmidFinder; ResFinder, https://genepi.food.dtu.dk/resfinder; Virulence Finder, https://cge.food.dtu.dk/services/VirulenceFinder).

APEC and extraintestinal pathogenic *E. coli* can share some virulence factors and plasmids associated with resistance (*10*–*12*). A previous study found that APEC and UPEC possess common virulence factors such as *papG II*, *iss*, and *ompT* genes (*13*), which encode an adhesin and 2 evasins related to colonization of the urinary tract and avoiding the host's immune system. Our results (Table) revealed that the RG299.1 strain carries genes associated with iron transport, invasion, adherence, and pathogenicity. The presence of those genes makes the strain potentially invasive and can contribute to other virulence traits such as biofilm formation (*14*). Genes involved in the biogenesis of capsules belonging to the capsular group II were also identified, such as *kpsD*, *kpsM*, and *kpsT.* Those genes are responsible for the transport of polysaccharide across the cytoplasmatic membrane and their structure in the cell surface (*14*).

## Conclusions

Six strains of *E. coli* ST131-H22 isolated from poultry with colibacillosis were identified in Brazil (*2*). Whole-genome sequencing and phylogenetic analysis revealed a high similarity between those strains and strains found in other countries, including strains of human origin, revealing a threat to public health. In wild birds, colibacillosis is a high risk, because the infection by APEC can rapidly progress from airsacculitis to sepsis, leading to animal death.

Our phylogenetic data revealed a close relationship between the strain from the current study and strains of veterinary origin isolated from a chicken (GenBank accession no. SRR14995942) characterizing a food source type, from a turkey (GenBank accession no. SRR7469899), and from a domestic *Gallus gallus* (junglefowl) (GenBank accession no. SRR7469899). The geographic origin of the ST131 strains could be heterogeneous; however, our results demonstrate a major similarity in the variations of this ST among isolates from different continents.

Our findings suggest that illegal animal trade can play a major role in the dissemination of APEC, considering the poor hygiene conditions to which the animals are subjected. The overcrowded wildlife triage centers that receive seized animals might have difficulty controlling pathogen transmission through biosecurity failures. Even though the principal route of contamination for pathogenic *E. coli* strains is through foodborne transmission, domesticated wild birds can potentially serve as an additional contamination route (*15*).

ST131 is becoming the prevalent extraintestinal pathogenic *E. coli* multidrug-resistant lineage worldwide, highlighting the necessity for monitoring. More studies are needed to investigate the presence of high-risk lineages of APEC in wild birds. A PCR triage to detect ST with pandemic potential is necessary in the routine care of birds. This monitoring becomes particularly critical when those birds are participating in a program to reintroduce and release. In addition, the next-generation sequencing of APEC varieties can clarify their origin and the relation between them, enabling better monitoring.
